# A Plea for Reform in Criminal Jurisprudence

**Published:** 1896-05

**Authors:** F. E. Daniel

**Affiliations:** Austin, Texas


### TEXAS MEDICAL JOURNAL.

ESTABLISHED JULY, 1885.

PUBLISHED MONTHLY.—SUBSCRIPTION $2.00 A YEAR.

VOL. XI. AUSTIN, MAY, 1896. NO. 11.

### Original Contributions.

### A PLEA FOR REFORM IN CRIMINAL JURISPRUDENCE.

BY F. E. DANIEL, M. D., AUSTIN, TEXAS.

[Read at the meeting of the Chicago Medico-Legal Society, January 12, 1896; revised and enlarged, and read before the Texas State Medical Association, at its 28th annual meeting, Fort Worth, Texas, April 28, 1896, and before the Section of Medical Jurisprudence and Psychology, American Medical Association, at Atlanta, Georgia, May 6, 1896.]

IN ALL ages and amongst all peoples, civilized and savage, so far as we have any record, the instinct of race, tribe, or national preservation has led them to regard the welfare and prosperity of the people as the supreme law. No individual interests were paramount to that of the people, and personal rights were curtailed, or sacrificed to the public good. For ages this has been formulated into the familiar maxim, *Salus Populi est Suprema Lex.* And as a corollary, it has been a maxim that "the few shall suffer, or be sacrificed (if need be), for the preservation or safety of the whole;" hence, the democratic doctrine, "the majority shall rule." Amongst primitive peoples, this principle was carried to the extent of destroying feeble or decrepit offspring who would become a burden or hindrance to the state or tribe; and in the interest, too, we must assume, of race integrity. Amongst tribes, the chief decided what was best for his people, and his word was the law, to which unhesitating obedience was exacted. Ancient nations assembled their wise men, who considered all sources of danger to the people, and, according to the lights before them, conscientiously guarded against them. It remained for a twentieth-century civilization, an enlightened republican government, to ignore this "supreme law,"—to give it a secondary place, and to make the protection of *property* the highest and dearest consideration; in the enactment of laws to utterly disregard the danger of race degeneration; to permit, nay, promote and accelerate the propagation of untold evils and dangers to society and the race, through the medium of heredity. That a government should—possessing the power and means to do so—prevent an increase in the criminal element, is a proposition which it would seem requires neither argument nor defense; that it should permit—nay, deliberately propagate and encourage an increase of criminals out of all proportion to population, is monstrous; it is hard to reconcile with ordinary common sense.

In light of the rapid and alarming increase of crime and criminals in this country, presently to be demonstrated, it is evident not only that there is something radically and fundamentally wrong in our system of criminal jurisprudence, and that our penal methods are a failure of the ostensible ends sought, but that reform has become an imperative, pressing and immediate necessity. It is demanded by every consideration of safety to society, public morals, public economy, and especially duty to tax-payers, who bear the burden, not only of this, but of every other class of defectives; leaving out of consideration the higher ground of humanity to the unfortunate victims of heredity and environment (for such are criminals for the most part), and omitting all reference to the claims of posterity to protection. What that burden is, may be faintly estimated when we reflect upon the expense incidental to the detection, arrest, prosecution and punishment of the vast hordes of criminals, together with the pay of the army of constabulary, police detectives, law officers, judiciary and prison officials, and the maintenance of prisoners; and an idea of the magnitude of the danger threatened and constantly augmented, may be gathered from statistics.

According to the last United States census (1890), there were, in 1850, 6,737 prisoners in the United States, or one to every 3,442 of the population. In 1890, there were 82,329 prisoners, or one to every 757 of population. In other words, while the population of the United States has, in four decades, increased

170 per cent., the prisoners have increased 445 per cent. And these were *prisoners*—in custody or under sentence, or awaiting trial. The number of criminals at large, evading arrest or unknown, is to be added to this,—something impossible to estimate, but a very large per cent., no doubt. At the present ratio of increase, it will be a matter of very short time when the criminal will outnumber all other elements of population.

Nothing could testify more emphatically to the inadequacy of our system to meet the requirements than these figures; nor appeal more forcibly for reform; nor could anything illustrate more fully the needs of prophylaxis against hereditary criminals.

With such facts before us, it behooves a rational people to inquire into the causes that lead to such disastrous results; to ascertain wherein lie the defects in our system of jurisprudence whereby these things are made possible; to ask what are the factors concerned in this production and great and rapid multiplication of this evil, and to seek, by every legitimate means, to arrest it.

Beyond doubt, heredity and environment are responsible for a large share of it; the laws regulating marriage are sadly deficient, and licensing the sale of liquor as a source of revenue to the state, is another evil, next in order of consequence and potency; and I believe it can be shown also that the *execution of our penal methods operates to contribute to the ever swelling hordes of criminals*, rather than to checking or diminishing it, as I will presently endeavor to show.

The system of criminal jurisprudence in this free and enlightened country appears to be founded on the sole idea of revenge, and *punishment* to be the end and object of all penal statutes. And this, too, under the claim and pretext that it is justice. Oh, justice, how many cruel wrongs are committed in thy name; to paraphrase Madame De Stael's apostrophe to liberty. I can not see that there is the remotest connection between punishment for crime, and justice; there is not an element of justice in it. If a man slay you, in what way are the demands of justice satisfied by his execution? What satisfaction is it to the widow and children left destitute by your death? Or, if a man fire my house, wherein is "justice" satisfied by sending him to prison to labor? In either case it is not justice—it is revenge; and who gave the state the right to take vengeance? Nor is "justice" the aggrieved party, it is I—or your family—who should be satisfied. Justice and equity are synonymous, and contemplate *restitution*, to make even,—amends; and the ends of justice would be better served were the murderer or incendiary stripped of his possessions for my benefit, or the benefit of those robbed by his hand. And where the State metes out so-called justice by punishing a man for crime committed under the influence of liquor, it is worse than a farce to call it justice; it is the rankest kind of injustice. The state licenses the sale of liquor, deriving revenue thereby. It thus aids and abets the saloon-keeper to tempt the young, the weak, the reckless and the unwary to put that into their stomach which robs them of reason for the time being, and deprives them of the power to resist an evil impulse. Murders have been committed unconsciously by young men under the mania of their first intoxication. The state hangs or imprisons that man, robs his wife and children—deprives them of their bread winner and of bread—and overwhelms them with disgrace,—maybe brings them on the hands of the tax-payer—as inmates of the poor farm, and calls it justice! What a cruel wrong! What a burlesque on justice! The justice in this case would seem to demand restitution to the imprisoned man for the destruction of his worldly prospects, for blighting his life, and the state is accessory before the fact;—and the one so put to death—the state should make restitution to his widow and orphans. The evil of the day and generation is the saloon.

But, so long as newspapers are run to make money, and not in the aid of humanity or science, or for the dissemination of truth, or, as is often the case, subsidized by the whisky ring, it were idle to preach against it; they will never aid science in any reform in the interest of truth, humanity or religion. No paper can be found with the honesty and independence to advocate any measure of reform—or to disseminate any truth in the interest of humanity that conflicts with that interest.

The failure of our system to either protect society or diminish crime is in a measure due to lack of vigorous enforcement of the laws; but principally it is due to defects inherent in the laws themselves.

Under the existing system, in order that crime may be appropriately *punished*, crimes are classfied and a penalty affixed to each. One great difficulty is that criminals are not classified also. The fact that a murder, for instance, was a first offense, cuts no figure. There is a penalty for murder (death by hanging) and all murderers, old and young, male and female, good family or bad, penitent or indifferent, first offense or fortieth, must expiate it—to satisfy the ends of justice.

There is a penalty for homicide, and all homicides must conform to the penalty; there is no qualifying circumstance, except *the degree of the offense.* It is simply left to the judge to determine the crime, classify it, and looking in the book, find the companion-piece to it, the penalty—and fit the one to the other; and to the jailor or sheriff to execute the penalty. There is no discrimination. The young boy for his first offense committed, it may be, in resentment of an insult, and from an impulse beyond control, or from fear of his life, or what, unfortunately is most frequently the case, while *under the influence of liquor;* or, if a theft, committed from want, or temptation, or what not, is thrown, first in jail to await trial. There he is surrounded by a vicious, brutalizing environment, huddled, perhaps, with a lot of filthy negroes and Mexicans, all hardened criminals; in fact, made to breathe an atmosphere fatal to every instinct of self-respect, and calculated to crush out every atom of manhood. When brought to trial the fact that it was his first killing, that he was drunk at the time, and for the first time in his life, and had not even a knowledge of what he had done; that, realizing the situation he is deeply penitent, and would give worlds to undo it and make restitution,—does not in the least qualify the offense, except, perhaps, it may secure for him the minimum term of imprisonment,—the lightest punishment *that goes with that kind of crime;* it is a definite term, and carries with it eternal disgrace, social and business ostracism and *disfranchisement.* Could anything be more unjust? Why, what would be thought of a doctor, for instance, who, having all his cases diagnosed for him, should treat every one of a class exactly alike, with the same dose? and without regard to age, sex, temperament or environment? Here is a case of fever: here is a formula for fever for all comers. Here is a case of rheumatism: here is the treatment for rheumatism for all ages, sizes, sex, color or "previous condition,"—*the book says so.* Failure would be a foregone conclusion. And so with our classification of crime, with its penalty attached, without a corresponding classification of the criminal; it is, as we have seen, a lamentable failure.

Again, by existing methods the State essays to purify the morals of society by perpetrating a shocking crime. The law says "thou shalt not kill," and forthwith gives us an object lesson in killing, and in cold blood!

The pretext for putting a man to death to protect society can only apply to the habitual or born criminal;—and I submit the ends can be accomplished by a means less revolting. Surely there could be no such pretext urged in a case—say like that of Dr. Jones, a man who had lived a life of usefulness till past 50, a respected citizen--prominent, indeed, in business and society. He had been president of this society. A circumstance occurred which so exasperated him that he felt compelled, to vindicate his honor, to take the life of the man who had injured him, as he thought. Although justified in his own mind, the law held it to be murder, and he was sentenced to death (a subsequent trial sentenced him to twenty years in the penitentiary). Because of this one act, would this man have been held to be a danger to society which must be eliminated? And even with regard to the natural criminal, is not the question of responsibility to be considered? Take Holmes, Guiteau or Prendergast, acknowledged dangers; they could no more change their nature than a leopard could change his spots; it was born in them to kill. Should they—morally insane, confessedly—be cruelly put to death for responding to the promptings of a natural predisposition? Why not lock them up securely, as we do man-eating wild beasts in captivity? Because they might or would kill if they had liberty, do we feel called upon to shoot them?

But heredity and environment, as potent as they are, and as prolific, are not the only factors of increase. I believe it can be demonstrated that the existing conditions and methods of our system not only fail of its ends, but operate to defeat them, and become as I have said above, a tributary to the growth of crime and the multiplication of criminals. Take an illustration, a case in point which I have in mind. A boy of 17, small for his age, a mere child,—was wanted for suspected complicity in a burglary. He came of respectable parentage, amongst whom crime was unknown; but owing to environment he grew up to be regarded as a bad boy. The policeman was afraid of him, and attempted to take him by strategem. He employed a chum of the lad to call him to the door at night, when the policeman sprang upon him out of the darkness, and without a word, covered him with a pistol. The boy, in fear of his life, doubtless, and by instinct of self-preservation, shot and killed the policeman. For this he was sentenced to seventeen years in the penitentiary at hard labor. At this writing he has served eight years; has a record of uniform good behavior,—has given every evidence of repentance and a desire to lead a correct life; the end and object of his incarceration has been accomplished; he is "reformed;" he has been *punished.* But no,—his sentence was for a definite term of years,—that's the law,—and he must serve nine years more, when, better or worse, he will be released, deprived of every right of citizenship, *sans* pride, *sans* hope, ambition or self-respect, his father's name dishonored, his widowed mother's heart broken,—the best years of his life, all of his youth spent in a felon's cell, what a mockery his "liberty" will be. What will he have to live for? Is it likely that he will become a moral, upright and useful man? Or, will he, feeling that he has been unjustly punished, *vengeance* thrice-fold taken upon him for an act which he has long since repented,—that the State is his enemy and mankind his natural foe, his hand, like Ishmael's, against every man, will he go to swell the ranks of the hardened and irreclaimable criminals? Who can ask?

This case will show the absurdity and the worse-than-uselessness of the "definite sentence" system. It makes criminals, rather than cures them.

The inadequacy of existing statutes to meet the requirements is an exceedingly grave matter, and should cause deep concern to all thinking and patriotic men. Not only that they fail to repress crime and protect society, and, as we have shown, operate to increase it, but the want of confidence on the part of the public engenders a feeling of insecurity which drives them to the commission of those acts of lawlessness for which they are so severely but unjustly censured. Self-defense and the protection of home are the strongest instincts of human nature. The people of Texas are as loyal and law-abiding as are to be found anywhere; but when they realize that the methods of dealing with the rapist and murderer, and the double crime, rape *and* murder (and that too, most frequently, of tender young children), are not effectual to put a stop to it, even when the law is swiftly executed, but, on the contrary that an execution, even in the horrid form of the stake, actually appears to *incite others to the crime*, it simply drives them to madness. The horrible execution of Henry Smith at Paris, must have been known to every negro in Texas; but it did not deter another negro from committing a similar outrage a short time after at Tyler, and he met a similar fate. Nor have the several prompt hangings for rape been attended with more salutary results. *Rape is notoriously on the increase*, not only in Texas, but in other States, and lynch law is brought into execution.

In support of the assertion that an execution incites others to crime instead of having a deterrent effect upon the evil disposed (such is the theory of our system—"to strike terror in his soul" and awe him into good behavior), I refer to statistics to show that in England, of 167 criminals condemned to death—a fact often quoted—all but three had witnessed executions. May this not be a pyschological problem not yet unraveled by medical science? Our knowledge of hypnotism is yet crude and imperfect. May it not be that persons—especially the ignorant—witnessing so shocking and impressive a sight, receive unconsciously, the "suggestion" to murder? What is it that prompts a person to do, against his will and intent, an act which he knows he should not do, and for which he will be speedily put to death? Poe calls it the "Imp of the Perverse."

But the worst feature connected with the subject, and that which drives the people to desperation is, almost as many offenders escape as are caught; and when they are caught, there are so many delays, appeals, writs-of-error, feigned insanity, etc., that the feeling of insecurity is intensified to the last degree, and the people take the matter in their own hands. They do not understand the reasons why, but they recognize the fact that the laws can not be depended upon for the suppression of crime and the protection of their families, and their acts are a spontaneous though very crude and primitive effort at a remedy.

I am inclined to believe that the fountain head and source whence flows this great evil—lynching—can be traced to the unwise policy that obtains of paying legislators day-laborers' wages. In Texas the per diem is $5; and after sixty days it is reduced to $2. It is hardly to be expected that such remuneration would command a very high order of law-making talent. Statutes enacted by men who can afford to leave home and business for $2 per day, who know nothing of the requirements of sanitary legislation, and don't want to be told, and could not fairly comprehend the subject if they were told, are apt to be defective, ambiguous and conflicting, and so afford grounds for endless "errors," protracted trials and tedious delays. With the exception of a respectable per cent. of really able men,—lawyers, for the most part—who have political aspirations or some reason for serving, other than the pittance of pay, and who really make a pecuniary sacrifice in so doing—the legislatures of many States are composed of average representative citizens, farmers, merchants, mechanics, or else, young "limbs-of-the-law;" and this class is in the majority. It is this element that defeats all attempts at reform; who ridicule the efforts of the medical profession to secure improvement in the medical-practice acts, and who mocked and insulted the noble Christian women of whom Mrs. Gardner tells us in the *Arena*, in their efforts to amend the "age of consent" statutes. It would be difficult, I apprehend, to make this sort realize that their ignorance, bigotry, or most of all, conceit that makes them refuse to listen to any suggestions of reform in accord with science, and intolerant of advice, is the real and first cause that leads to lynchings. They are loud to denounce it, and without a suspicion of the truth, they serenely set about to enact statutes *to punish the lynchers.*

Indeed, may it not be that herein lies the one great cause of the inadequacy of our system? In the enactment of the criminal and health statutes the requisite knowledge is not brought to bear. The making of all our laws is in the hands of men who make no pretensions to science, and they are notoriously averse to being advised.

Science is applied knowledge. The most civilized races are the most scientific and progressive. We live in an age of enlightenment and advanced civilization. Never, at any period of the world's history, have the facilities for the acquisition of knowledge and the dissemination of information been so great in every department of life, and yet, in many respects, and especially in that pointed out above,—vital to society and human happiness and well being, man fails to profit by experience and neglects to make use of the knowledge gained.

Knowledge, acquired in whatever way, is applied in the various arts, and made subservient to man's wants. In enlightened governments there are heads of departments whose function it is to gather and formulate the knowledge bearing upon their respective interests. This is true of everything except man's most vital interests—his health and well being, and the preservation of a healthy standard of race. In this great country there is no department of public health, and State medicine is a nullity. The vast store of knowledge gleaned by laborers in the field, is not utilized in framing laws for the protection of the public health and morals and race preservation.

On this head Judge Benjamin Abbott, in apologizing for the jurists and the want of progress in the jurisprudence of insanity (Ref. Bk. Med. Sci., page 122), says: "The rude division into 'idiots' and 'lunatics' of two centuries ago survives in jurisprudence to-day. . . . Jurisprudence has had no peculiar methods of studying the subject, but has been accustomed to follow the course of medical science, and to accept, sometimes, indeed, only after long hesitation and inquiry, the results which skillful and experienced alienists have united in declaring established."

Jurisprudence has not studied the subject, yet will not accept the conclusions of those who have; here's a confession of bigotry and intolerance.

Judge Abbott here uttered a pregnant truth, one which has a wider application, perhaps, than he was aware. It is the key to the problem, why our criminal and insanity laws are a failure.

To confess that the jurisprudence of insanity has not been revised in two hundred years, because jurists will not accept the conclusions of scientific investigators in this field, when in the meantime insanity has been studied in all its phases, and subdivided and classified till now there are nine forms of idiocy and six forms of madness known to alienists, would indicate that one or other of the forms of idiocy or mania had seized upon our rulers and law makers. Our statutes belong to past ages.

Granting that the system is a failure, wrong in conception, too narrow in scope, and ineffective for the ends sought to accomplish, what should be done to remedy it?

The thinking members of the medical profession are being rapidly converted to the belief that *crime is a disease;* that habitual criminals are *sick persons*, and that their condition calls for a more enlightened method of management. They know that many, if not most of them, are subjects of hereditary transmission of vicious temperaments, and are victims of vicious environment. It is beginning to be apparent that sooner or later a change must come, precisely such change as has taken place with insanity and inebriety. It has not been very long ago that drunkenness was regarded as a criminal offense, or rather, as a misdemeanor,—and was punished; nor since the insane were regarded as monsters,—or "possessed of a devil," that was to be exorcised only by blows and by straight-jackets; nor since the poor dement or idiot and the harmless maniac were, by law, burned as "witches," an act for which civilization is not yet done blushing. Opinion has changed radically, as regards these unfortunates; and with it, the system of dealing with them. The insane, and in some States, the inebriate now find repose and tender care and rational treatment in the great eleemosynary institutions of an enlightened age; and so opinion is fast changing with reference to crime and criminals, and the mission of science is to bring about a corresponding change in their status and management. Professor Flint, in his paper, says (*N. Y. Med. Journal*, Feb. 15, 1896):

"Crime is a disease of our social organization. It is true that it is ineradicable, but it may be restricted within much narrower limits than at present exist. Crime calls for intelligent and scientific treatment. While crime can not be abolished, all criminals are not hopelessly affected with crime. . . . Crime may be a constitutional disease, as in the born criminal, or it may be due, in individual cases, to surroundings, teaching, or example—a sort of contagion. It has been abundantly shown that criminals may be divided into two great classes, the curable and the incurable; but the disease which we call crime has nearly as many phases and varieties as are presented by the nosological catalogue. Society needs the aid of competent men to undertake the task of separating the curable from the incurable,—to restore the former to usefulness, and to protect our social organization against the latter. Jurists,—so-called lawgivers, and those who execute the law,—have failed. In my opinion, the only hope is in the medical profession."

The problem is, *how can these views be impressed upon those who are entrusted with the enactment of the statutes?* The utter failure of the State Medical Association in Texas to awaken in the minds of legislators a just appreciation of the dangers attending the indiscriminate practice of medicine, or the unrestricted sale of nostrums deleterious to the public health, gives but little assurance that anything the medical profession of this State might bring before them upon this great subject, would receive more respectful consideration.

State medicine is the application in the aggregate, of the principles of medical and sanitary science to the prevention, cure, mitigation or relief of evils which affect the social body, and the prevention of those evils to posterity. It bears the same relation to the State or society that the individual physician bears to his clientelle; and embraces, of course, measures of prophylaxis against future ills as against existing evils. For illustration, any measure calculated to improve the race,—we will say, —restrictions upon marriage limiting the privilege to the fit, or castration of natural criminals, or insane criminals, or the criminal insane, to cut off succession as here advocated, are as much within the scope of its beneficent functions as is quarantine against disease; indeed, the entire treatment of criminals, as hereinafter proposed and indicated, comes most appropriately within its province; and when we shall have succeeded in getting a department of public health, the first step will have been accomplished. The State owes no higher duty to posterity than to protect it against a multiplication of those evils we now deplore, and are ineffectually battling against.

Medicine has ever been characterized by humanity and benevolence. The profession do all in their power to relieve suffering. Our grand hospitals and asylums are monuments to the benevolence and unselfishness of medicine. Yet it seems to be, after all, a false philanthropy, as it enables the afflicted ones to live on and beget more children for the next generation to care for. Thus evil comes out of good, and our best intentions react to the ultimate detriment of society. By practical charity, alms giving, and the tender care for the defectives and diseased, the operation of nature's laws is defeated, and the unfit survive,—and breed and multiply like flies.

On the subject of marriage, Judge C. H. Reeves, of Plymouth, Indiana, in a work called "The Prison Question," the most logical exposition of the subject I have ever seen, says:

"In regulating marriage, the law says that none shall marry within the third degree of consanguinity, and in some States the fourth, because marriage between near blood relations is likely to produce offspring deformed or diseased, physically and mentally. Insane and idiots shall not marry, because they can not make a contract, and because of hereditary tendency to produce idiots and insanity. It makes it a crime to marry in any of these cases. In this it aims to prevent degenerate offspring and protect individuals and society against the evils that would attend such offspring. * * *

"But if the vilest mortal that can live—one not in these classes—sees proper to marry, the law issues the license for the asking, taking a fee, makes a record, and leaves the offspring and society to shift for themselves in the best way they can. The confirmed inebriate, the weak-minded and semi-idiotic, the confirmed criminal, the offspring of the half-witted and insane, if lucid at the time,—the incurably diseased, the scrofulitic, the syphilitic, the hereditary pauper, the depraved and reckless—even paupers while in the poorhouse, and criminals while in jail, are in every way encouraged, given license, and are protected by the law. No thought is taken for the unfortunate offspring, nor for the body politic or social, and the irreparable evils that must fall upon all. The church adds its sanction, and its ministers aid in making these civil contracts, by performing the ceremony with benediction and prayer. * * *

"If it is wise to prohibit polygamy, marriage between near relations, between the insane and idiotic, because of heredity and transmissions of evils, it is equally wise to prohibit it in all cases where like evils may follow. If the law has the power to prohibit and punish violations in one case, it has equal right in all others.

"There is an endless procession of children from all these sources coming into the mass of population to live lives of crime, immorality, want, suffering, misfortune and degradation, transmitting the taint in constantly ever widening streams, generation after generation, with the ultimate certainty of the deterioration of the race, and final irreparable degeneracy.

"It seems to me that there is a moral obliquity that affects the entire mass of political, social and religious leaders and teachers on the subject here being considered. When we analyze the views and actions throughout, the glaring inconsistency and unreasonableness that seems to fill them has no parallel in any other matter seriously affecting individual and the public welfare. Among the first is a false modesty, that is shocked by any allusions to the most evident and debasing facts that stare everybody in the face on all sides; that rub everybody at every turn.

"The church devotes its time and energies to prove that every human body possesses an immortal spiritual body, that is liable to future torture unless it be made perfect in morals and truth, and that must be done while it remains in its mortal shell. It pleads and raves for prohibition of liquors and tobacco, for forced observance of Sunday, for forced attendance on schools, for recognition of God, Christ and the Protestant religion in the civil constitutions, and for sundry other restraints and commands, with penalties, in order to save these imperiled souls. Reformers go about the land devising ways and means to educate, civilize, provide for and elevate the ignorant, the degraded, the poverty stricken that pervade every plane of human action, and wander in and out among the people everywhere. And yet these, with general society added, hold up their hands before their faces in horror, if some honest soul who has truth for a guide, calls to them to look, and points them to the source of the evils they are battling with and tells them they are responsible for it all, for the law is only their united will in statutory phraseology. That it is the result of their voluntary blindness and false conception of civil, moral and religious duties. That they are seeking to deal with evil conditions alone, instead of the causes of them, and while trying to mitigate the evils in the results, are supporting, increasing and enlarging the causes. That on every other plane of action they recognize and deal with the causes; but with men and women they ignore the causes and battle with results alone. That they regard domestic brutes as of more importance than they do human beings."

Doubtless Macaulay had this condition of society in mind when, forty years ago, he predicted the disintegration and downfall of the American Republic. Writing to Henry S. Randall, in 1857, he said: "I have long been convinced that institutions, purely democratic, must, sooner or later, destroy liberty or civilization, or both. Your constitution is all sail and no anchor. Either some Cæsar or Napoleon will seize the reins of government with a strong hand, or your republic will be as fearfully plundered and laid waste by barbarians in the twentieth century as the Roman empire was in the fifth, with this difference—that the Huns and Vandals, who ravaged the Roman Empire, came from without, and that your Huns and Vandals will have been engendered within your own country, by your own institutions."

It would seem that a rational people, with such facts before them, for instance, as those furnished by Dugdale's history of the Jukes family, from whom 1200 criminals descended, would profit by it, and take steps to close the flood gates of evil. And the Jukes case is not an exceptional one, by any means; there are thousands such; they exist every day, everywhere.

The magnitude of the evil and danger resulting from our criminally lax marriage laws, is simply appalling. Yet few ordinary citizens—those who pay the taxes—have a conception of it, or realize the extent of the cruel wrong done them by permitting it.

An intelligent comprehension of the subject would, therefore, indicate that the first step in needed reform is state regulation of marriage with a view to the arrest of descent of crime by hereditary transmission of the tendency. And, dealing directly, then, with the crop on hand, it is suggested that punishment, as such, as a penalty, should have no place in a civilized code. It is permissible only as a feature of discipline incidental to reform; that as criminals are divisable into the two great classes, the curable or accidental criminal, and the habitual or incurable criminal of Lombroso,—the end and object of penal enactments should be the *cure* of the *curable*—the reclamation to usefulness of those who are amenable to it, and the *elimination of the in-*

*curable*. To this end, therefore, a classification of all criminals is necessary.

Classification can only be done by medical men. The entire subject comes legitimately within the scope of State medicine; here, indeed, it finds its most appropriate field.

When the character of the crime has been determined by the court, it would seem to be in accord with the requirements of the case and the dictates of an enlightened humanity, that there should be medical men to *diagnose the criminal*, and prescribe the course, which, in their judgment, is best calculated to meet the demands. If it be one of the curable class, the treatment, consisting of restraint, discipline, hygiene, education, environment and healthy labor, should be such as to induce a determination to never offend again. Pride and self-respect should be fostered, for they are the highest incentives in life to good behavior. The term of imprisonment should depend upon the progress made in reformation; on good behavior;—the culprit made to realize that when he gives evidence of fitness to be trusted with his liberty, it will be restored to him. And primarily he should be lifted above the environment calculated to debase him in his own mind. The incurable,—the born criminal of Lombroso,—should be dealt with as a permanent enemy to society, and the first aim in his case, after sequestration, should be precautions against a progeny; to cut off his race. When a man has been diagnosed as a natural, i. e., an irreclaimable criminal, twice convicted of any felony, along with the forfeiture of liberty for life, and all other rights, he certainly should be deprived of the right (and the power, should chance permit) to inflict a progeny upon the next generation. Why *not?* Can anyone give a single reason why this right should be respected when all others are taken away? I think not. The strange veneration people seem to have for *those particular possessions*, which induces them to plead that they be spared even when every other right has been forfeited and taken away, is the last remnant of the old Phallus worship; a superstition of the fifth century.

Capital punishment is becoming more and more abhorrent to thinking people, and is being very generally condemned by medical writers as *barbarous, useless and unjustifiable*; and castration as a substitute therefor, is rapidly growing in popular favor. Much of the prejudice that existed against castration is disappeariing under the light of reason. Indeed, it seems to me that there is every reason why capital punishment should be abolished, and isolation and emasculation substituted; and the fundamental principles of justice demand that where possible, restitution should take the place of imprisonment. It is true, privation of one's liberty might be called punishment, it is so, incidentally; but let it be done for the purposes of reformation and for the improvement of the morals of society and not of revenge—miscalled justice. Coporeal punishment never made a school boy good; and the morals of a community can never be purified by a system of punishment entailing eternal disgrace as penalty for misdeeds. The sense of injustice arouses resentment and stirs the worst element in one's nature.

Gentlemen, the time has come and the occasion demands—if we would make an effort to preserve the integrity of our race and the safety of the republic, when the medical profession must look at this question from the higher standpoint of guardians of society and conservators of the public wellbeing, and none the less as trustees for posterity. It should be insisted that the voice of science be heard; that the great truths revealed by study and research, by laborious investigation, experimentation and compilation—truths vital to the dearest interests of mankind should be utilized in medical and criminal jurisprudence. Our entire system needs to be recast along broader lines, and made more comprehensive; remodeled and adapted to the changed conditions of a 20th century civilization. As at present constituted it deals with results alone, and utterly ignores causes. We concern ourselves with, and can not solve the problem of what to do with the criminals of this day and generation—without a thought toward, or an effort to close the avenues through which pour in ever swelling tides of the evil we vainly attempt to remedy.

Sisyphus, condemned to eternally roll the stone, had no more hopeless, endless task than we are now engaged in; nor the Danaides one more impossible of accomplishment; it is as irrational as the attempt to purify a sewer by throwing disinfectants into the outlet. The helpless, worthless, vicious and dangerous come faster than love, philanthropy, religion, science and law can cure, care for, reform, or dispose of them.

Doubtless, by an organized effort on the part of the two learned professions, medicine and law, Congress can be awakened to the necessity of taking steps to make available sanitary knowledge in the jurisprudence of medicine and crime; to create a department of public health and hygiene, whereby such knowledge can be disseminated and made to reach and influence legislators, however unwilling.

While we may never be civilized up to a system of scientific breeding of peoples (as we do our stock), it unquestionably lies within the scope and power of State medicine to eliminate much that is evil, and bring about great improvement, even in the next generation, in the physical, moral and intellectual status of society. And chief amongst the agencies effective to this end will be,—State regulation of marriage, and sterilization. This is the mission of rational medicine; to the accomplishment of which the profession shonld address itself, with the conviction that duty requires it,—true philanthropy dictates it, policy suggests it, and it is demauded by every consideration of justice, mercy, and humanity.

